# Drift Suppression of Solution-Gated Graphene Field-Effect Transistors by Cation Doping for Sensing Platforms

**DOI:** 10.3390/s21227455

**Published:** 2021-11-10

**Authors:** Naruto Miyakawa, Ayumi Shinagawa, Yasuko Kajiwara, Shota Ushiba, Takao Ono, Yasushi Kanai, Shinsuke Tani, Masahiko Kimura, Kazuhiko Matsumoto

**Affiliations:** 1 Murata Manufacturing Co., Ltd., 1-10-1 Higashikotari, Nagaokakyo-shi 617-8555, Kyoto, Japan; ayumi.shinagawa@murata.com (A.S.); yasuko.kajiwara@murata.com (Y.K.); shinsuke_tani@murata.com (S.T.); mkimura@murata.com (M.K.); 2 The Institute of Scientific and Industrial Research, Osaka University, Ibaraki 567-0047, Osaka, Japan; t-ono@sanken.osaka-u.ac.jp (T.O.); kanai@sanken.osaka-u.ac.jp (Y.K.); k-matsumoto@sanken.osaka-u.ac.jp (K.M.); 3 JST, PRESTO, 4-1-8 Honcho, Kawaguchi 332-0012, Saitama, Japan

**Keywords:** graphene field effect transistors, drift suppression, sensor drift, biosensors

## Abstract

Solution-gated graphene field-effect transistors (SG-GFETs) provide an ideal platform for sensing biomolecules owing to their high electron/hole mobilities and 2D nature. However, the transfer curve often drifts in an electrolyte solution during measurements, making it difficult to accurately estimate the analyte concentration. One possible reason for this drift is that p-doping of GFETs is gradually countered by cations in the solution, because the cations can permeate into the polymer residue and/or between graphene and SiO_2_ substrates. Therefore, we propose doping sufficient cations to counter p-doping of GFETs prior to the measurements. For the pre-treatment, GFETs were immersed in a 15 mM sodium chloride aqueous solution for 25 h. The pretreated GFETs showed that the charge neutrality point (CNP) drifted by less than 3 mV during 1 h of measurement in a phosphate buffer, while the non-treated GFETs showed that the CNP was severely drifted by approximately 50 mV, demonstrating a 96% reduction of the drift by the pre-treatment. X-ray photoelectron spectroscopy analysis revealed the accumulation of sodium ions in the GFETs through pre-treatment. Our method is useful for suppressing drift, thus allowing accurate estimation of the target analyte concentration.

## 1. Introduction

**Figure 1:**
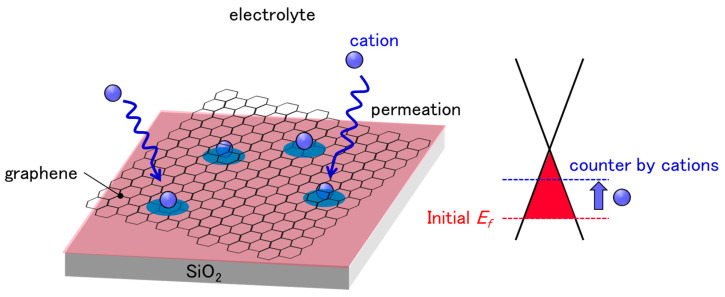
Schematic illustration of cation permeation into polymer residue and/or between graphene and SiO_2_ substrates. Graphene is initially p-doped by the effect of PMMA residue and SiO_2_ substrate. The diffusion of cations, which have n-doping effects to graphene, has a counter-effect on initial p-doping, resulting in CNP drifts.

The demand for sensing analytes in aqueous environments is rapidly increasing in various fields, including life sciences [1], environmental monitoring [2], and food safety [3]. Solution-gated field-effect transistors (SG-FETs), also known as ion-sensitive FETs (ISFETs), are promising candidates with which to meet this demand; thus, they have attracted considerable attention since their invention in the 1970s [4]. In particular, graphene is an ideal channel material owing to its high electron/hole mobilities [5,6] and 2D nature [7,8]. Moreover, substantial efforts have been made to produce chemical vapor deposition (CVD)-grown graphene, which makes large-area and high-quality graphene sheets available to everyone [9]. Therefore, solution-gated graphene FETs (SG-GFETs) have achieved considerable high sensitivity, and can be employed for the detection of analytes in solutions, such as ions [10,11,12], proteins [13], viruses [14], and bacteria [15,16]. However, SG-GFETs feature an inherent drawback, known as sensor drift or baseline drift, in ISFETs and SG-GFETs. Some studies have shown that the drift of ISFETs as well as SG-GFETs continue for more than 1 h [17,18,19]. Various models have been proposed and studied extensively to describe the drift in ISFETs, which include the diffusion of ions from electrolyte solutions into the oxide layers [20,21]. However, in most SG-GFETs, the graphene channels directly contact an electrolyte solution, that is, no oxide covers the graphene channels. Therefore, the established theories on ISFETs cannot be applied to SG-GFETs; thus, the drift in GFETs should be studied further. One possible reason for this drift is that the p-doping of GFETs is countered by cations in the solution, since the cations can permeate into the polymer residue and/or between graphene and SiO_2_ substrates. Here, we propose a method to suppress the drift, in which sufficient cations are doped to counter the p-doping of GFETs prior to the measurements, as shown in Figure 1. As a pre-treatment, GFETs were immersed in 15 mM sodium chloride (NaCl) aqueous solution for 25 h. The pretreated GFETs showed that the charge neutrality point (CNP) drifted by less than 3 mV during a 1 h measurement in a phosphate buffer. Our method is useful for suppressing drift, which enables the accurate estimation of the concentration of target analytes.

## 2. Materials and Methods

Graphene films were grown on Cu foils using a CVD method. A poly(methyl methacrylate) (PMMA) solution was coated onto the graphene/Cu foils. The Cu foils were etched away using an ammonium persulfate solution. The graphene films were transferred onto Si/SiO_2_ substrates with a thickness of 525 μm/290 nm. A source/drain electrode of 10 nm Ti and 90 nm Au was formed using electron-beam physical vapor deposition. Graphene channels were formed by oxygen plasma etching. After etching, the PMMA coat on the graphene was removed using an organic solvent. Thereafter, the graphene was annealed at 300 °C in an Ar/H_2_ atmosphere for 1 h to reduce organic residue.

**Figure 2:**
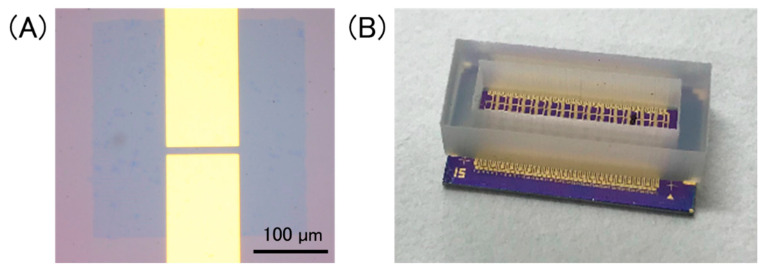
(**A**) Optical image of a GFET, in which a patterned graphene sheet is bridged between source/drain electrodes. Scale bar is 100 µm; (**B**) a photo of a device chip. A silicone rubber container was set onto the chip to hold a solution on GFETs.

Figure 2A shows an optical image of a GFET. The channel length and width were 10 μm and 100 μm, respectively. Multiplex GFETs were fabricated using the same substrate. A silicone rubber container was attached onto the GFET-array chip to hold an electrolyte solution (Figure 2B).

The electrical measurements were conducted as follows: The GFET arrays were immersed in 0.1× D-PBS(-) (Nacalai Tesque, Inc., Tokyo, Japan). A bias voltage (*V*_DS_) of 0.1 V was applied between a source/drain electrode, and a top-gate voltage (*V*_GS_) was applied through the electrolyte solution using an Ag/AgCl electrode. While sweeping the *V*_GS_ in the positive direction, the drain current (*I*_DS_) was measured using a semiconductor parameter analyzer (Keysight Technologies, B1500A). The CNP, where *V*_GS_ was applied at the minimum *I*_DS_ in the transfer curve, was calculated using polynomial fitting. We selected D-PBS(-) as the electrolyte owing to its suitability for biological applications.

Measurement with X-ray photoelectron spectroscopy (XPS) (ULVAC-PHI, Inc. VersaProbe) was used to verify the permeation of cations to the GFETs. The measurement spot is located at the center of the channel region. The Na1s and C1s peaks were analyzed to assess the cation accumulation and graphene quality, respectively. The baseline was subtracted, and the signal intensity was normalized to the peak area of C1s.

## 3. Results and Discussion

### 3.1. Transfer Curve Variability and Drifts of Transfer Curves

**Figure 3:**
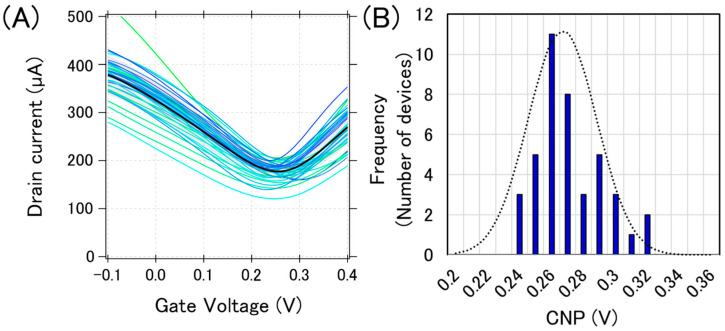
Initial electric characteristics of GFETs. (**A**) 40 GFET transfer curves from measurements (blue or green lines) and the calculated average curve (black bolded line) (**B**) The histogram of the CNPs with a Gaussian distribution fitting (black dotted line).

Figure 3A shows the typical transfer curves measured from a chip, where 40 GFETs were integrated. The bold black line in Figure 3A is the calculated average transfer curve. There are three types of variations in electrical characteristics: (i) current variation, (ii) CNP variation, and (iii) transconductance variation. These variations in the transfer curves may be attributed to the graphene tearing, PMMA residue on the graphene, and the impurity charge from the substrate. We previously reported that graphene tearing increases the resistance and decreases the transconductance [22]. It was also reported that both the PMMA residue and SiO_2_ substrate have a p-doping effect on graphene [23,24,25]. Moreover, water molecules also have a p-doping effect on graphene [26]. Therefore, our GFETs are expected to be p-doped, and it was proven that the observed CNPs were positive, as shown in Figure 3.

We used CNP as a representative feature value for a GFET because the sensing mechanism in GFETs is often demonstrated by the gating effect, which appears as the CNP shift [27]. Each CNP for the transfer curves in Figure 3A was calculated, and a histogram with a Gaussian distribution fit is displayed in Figure 3B. The calculated average and standard deviation of CNPs from 40 GFETs was calculated as 267 ± 21 mV, which are typical values for the initial state of GFETs under the measurement conditions used.

**Figure 4:**
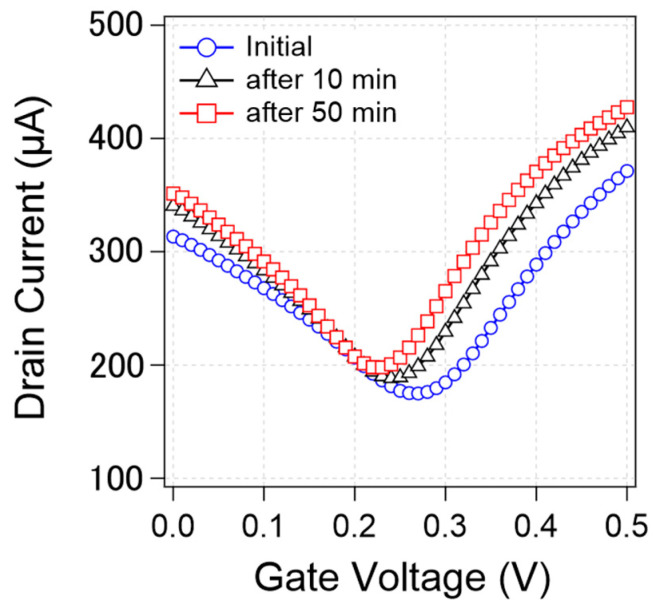
Change of transfer curves from a specific device during measurements. Initial transfer curve (blue circle) and the transfer curve after 10 min measurement (black triangle) and 50 min measurement (red square).

We found that the transfer curve was not stable but rather changed during the measurements. Figure 4 shows that the transfer curves from a specific device changed during the continuous measurements. As can be observed, the CNP, current value, and transconductance changed over time. In this study, we focused on CNP changes as a representative characteristic.

**Figure 5:**
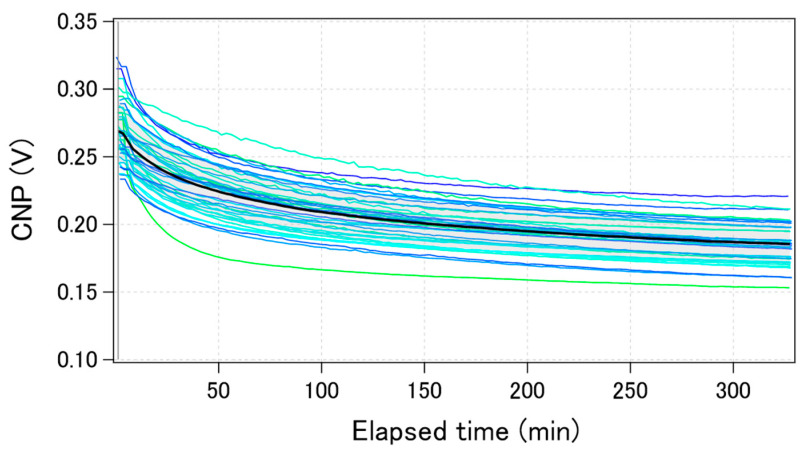
Time-course of the calculated CNPs during electric measurements for the same 40 GFETs in Figure 3. The black bolded line shows the calculated average value.

Figure 5 shows the time-course change of the calculated CNPs for the same 40 GFETs as in Figure 3 during electric measurements performed in 0.1 × D-PBS(-). The change in the calculated average CNP is also shown by the black bold line. The CNPs shifted by more than 50 mV towards the negative direction after 5 h of measurement. The trend was qualitatively the same among the GFETs, although the shift amounts were slightly different for each. The drift was initially large, but gradually decreased over time. Some reports have shown that when GFETs are used as biosensors or ion sensors, the CNP shift induced by target molecules ranges from a few to hundreds of mV [27,28,29,30]. Therefore, the drift of the CNP should be suppressed to less than a few mV to accurately estimate the concentration of analytes. To suppress the CNP drift, we investigated the factors that cause CNP drift.

### 3.2. Intermittent Electrical Measurements to Evaluate the Electrical Effects on the CNP Drift

Because the CNP drift was observed during the electrical measurements performed in an electrolyte solution containing D-PBS(-), it was expected that the drift was derived from electrical effects and/or surrounding effects induced by water molecules or ions. To clarify whether the CNP drift was induced by electrical effects, we conducted electrical measurements of the GFETs with a time interval. First, the GFETs were immersed in 0.1× D-PBS(-), and then the electrical measurements were conducted for 1 h. Subsequently, the GFETs were left electrically floated for 1.5 h; that is, all the input voltages from the gate and source/drain electrodes were stopped. Thereafter, the measurements were conducted again for 1 h; in the same manner, the GFETs were electrically floated for 1.5 h. Finally, the measurements were performed for 1 h. The average CNP was calculated from the data acquired from several GFETs on the same chip.

**Figure 6:**
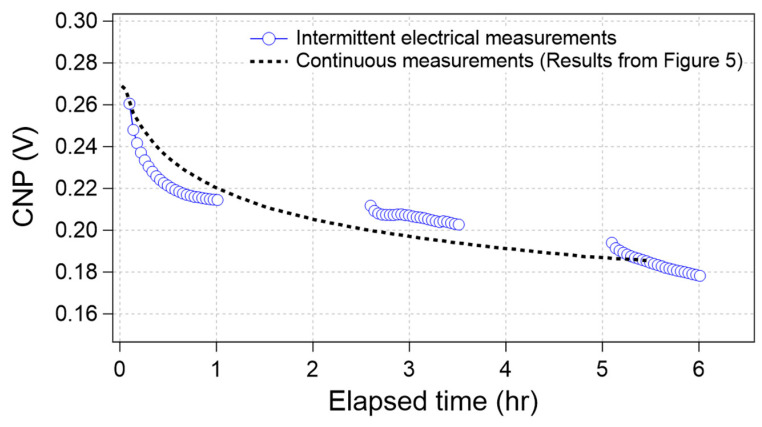
Time-course of the CNP value during intermittent electrical measurements (blue circle) and continuous measurements (results from Figure 5, black broken line).

Figure 6 shows the time course of the CNP obtained from the intermittent electrical measurements (blue circle). For comparison, the average curve in Figure 5, which was obtained through continuous measurements, also overlapped in Figure 6 (broken line). The results indicate that both plots seem to display the same trend, even though the measurement conditions were different. If the drift was caused by electrical effects alone, the two plots were separated because the effective elapsed time under the electrical measurements was 50% for the intermittent electrical measurement case. Therefore, we concluded that the CNP drifted even without electrical measurements.

### 3.3. Effects of Pre-Treatments on the CNP Drift

**Figure 7:**
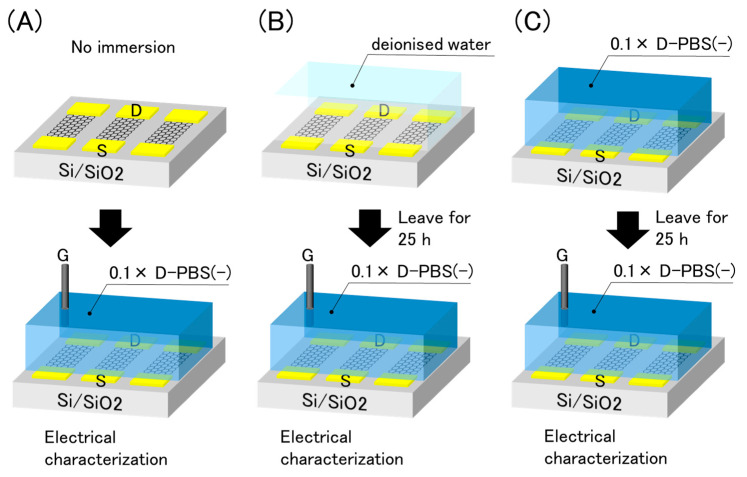
Schematic illustration of pre-treatments and electrical evaluation. (**A**) No immersion (**B**) immersion in deionised water (**C**) immersion in 0.1× D-PBS(-).

Because the drift was not affected by the electrical effects, we inferred that the electrolyte solutions were responsible for the drift. Put simply, the water molecules and/or ions in contact with the graphene were attributed to the drift. As the effect may differ depending on the solution, we immersed the GFETs into two different solutions, with and without ions, prior to the electrical measurements. The experimental procedure is illustrated in Figure 7. The GFETs were first immersed in deionized water or 0.1 × D-PBS(-) for 25 h as pre-treatments. Subsequently, the solution used in the pre-treatments was replaced with 0.1 × D-PBS(-), and electrical measurements were conducted. The results were compared with the initial properties, as shown in Figure 3.

**Figure 8:**
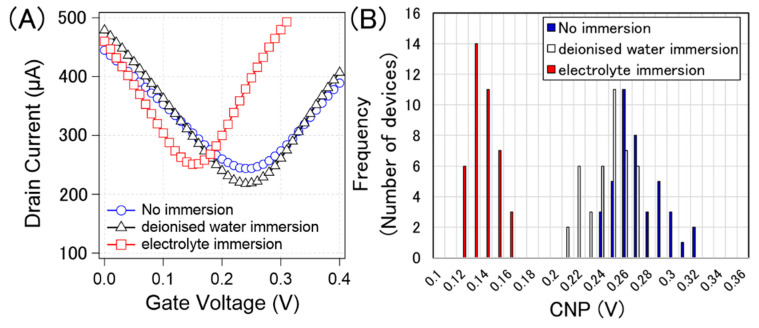
(**A**) Measured typical transfer curve of specific GFETs with no immersion (blue circle), immersion in deionized water (black triangle), and immersion in 0.1× D-PBS(-) (red square). (**B**) histogram of the CNPs from 40 GFETs with no immersion (blue), immersion in deionised water (white), and immersion in 0.1× D-PBS(-) (red).

Figure 8A shows typical examples of the transfer curves without pre-treatment, after deionized water immersion, and after electrolyte immersion (0.1 × D-PBS(-)). Figure 8B shows the histograms of the CNPs corresponding to each process. The calculated average and standard deviation of the CNPs from 40 devices for water immersion, electrolyte immersion, and non-treatment were 267 ± 21 mV, 240 ± 17 mV, and 132 ± 11 mV, respectively. Figure 8 indicates that the pre-treatments were effective in suppressing p-doping. It is known that water molecules are slowly intercalated between graphene and substrates [31,32]. This intercalation may potentially weaken the p-doping from the substrate, resulting in slight n-doping by water immersion. Although the effect of water immersion was not certain, the effect of electrolyte immersion was apparent n-doping on the GFETs. The results indicate that the ions were the main n-dopants. Jia et al. demonstrated that ions slowly penetrated between graphene and substrates, and graphene was gradually n-doped over time [33]. This phenomenon should also occur in the proposed system.

**Figure 9:**
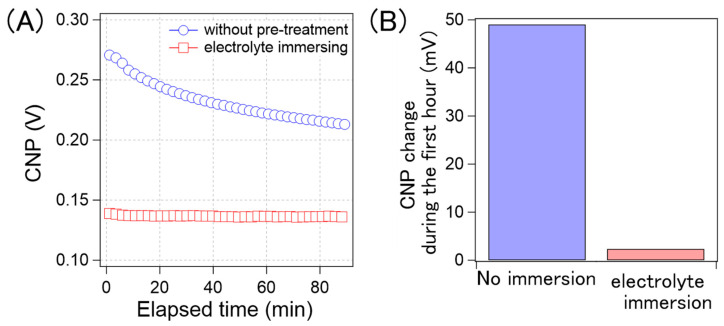
(**A**) Time-course of the calculated average CNP of the GFETs without the pre-treatment (blue circle) and with the electrolyte immersing (red square); (**B**) the CNP shift amount of the GFETs without the pre-treatment and with the electrolyte immersing during the first hour.

Based on these results, we developed a method to suppress CNP drift. If the CNP drift arises from gradual ion penetration, the GFETs, where ions are sufficiently penetrated prior to the measurements, should be stable during long-term electrical measurements. To verify this concept, we evaluated the CNP drift of GFETs immersed in 0.1× D-PBS(-) for 25 h and compared it with that of the non-treated samples. Figure 9A shows the time course of the average CNP values from the GFETs with and without pre-treatment. As can be observed, for the GFETs with pre-treatment, the CNP values were stable, but these values changed gradually for the GFETs without pre-treatment; this is because, as the CNP changes rapidly, the rate of change decreases over time. We believe that this rate correlates with that of the ion diffusion into the GFET. As the ions are accumulated in the GFET, the diffusion rate of the ions decreases. As such, the rate of change of CNP is thought to decrease. Figure 9B shows a comparison of the amount of CNP that changed during the first hour. The CNP for the sample without pre-treatment drifted by 49 mV in the first hour. However, the drift was reduced to 2 mV in the pre-treated sample. The results clearly indicate that the electrolyte immersion pre-treatment was effective in suppressing the CNP drift by 96%.

### 3.4. Types of Ions Affecting CNP Drift

Although we demonstrated that pre-immersion in the electrolyte solution suppressed the CNP drift, it still remains unclear which ions in D-PBS(-) account for drift suppression. It is known that cations, including K^+^, Na^+^, and Ca^2+^, are adsorbed onto graphene via cation-π interactions, leading to the same n-doping effect on graphene [33,34]. Therefore, we presumed that the cations in the electrolyte solution may have a significant impact on the CNP drift. To confirm this effect, we designed an experiment. We prepared GFETs immersed in a 15 mM NaCl aqueous solution for 50 h, which was used as a cation-containing solution. For comparison, other GFETs were immersed in a 15 mM HCl aqueous solution, which was used as a cation-free solution. After immersion in each solution, the solutions were replaced with 0.1 × D-PBS(-), and the drift characteristics of the CNP were evaluated.

**Figure 10:**
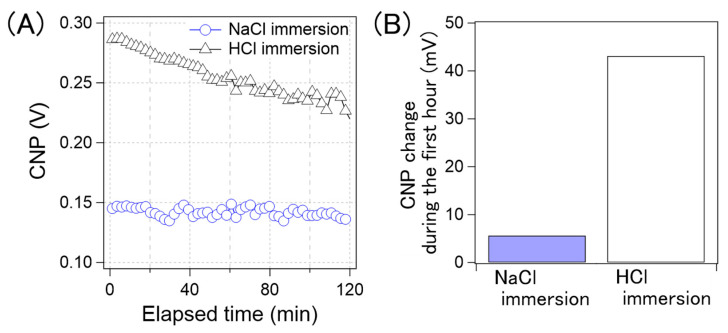
(**A**) Time-course of the CNP average of GFETs with NaCl immersing (blue circle) and with HCl immersing (black triangle). (**B**) the CNP shift amount of the GFETs with NaCl immersion and with HCl immersion during the first hour.

Figure 10A shows the CNP drift of the GFET immersed in the NaCl aqueous solution and aqueous HCl solution. Figure 10B shows a comparison of the amount of CNP drift in the first hour. The drift for the GFETs with the HCl aqueous solution immersion was 43 mV, while that with the NaCl solution was 5 mV. As can be observed in Figure 10, the CNP drift was significantly reduced by immersing GFETs in the NaCl aqueous solution, namely the cation-containing solution. Therefore, we concluded that the cations played an important role in suppressing the drift.

### 3.5. X-ray Photoelectron Spectroscopy (XPS) Analysis to Verify Cation Accumulation

Based on the effect of pre-treatment on the CNP drift, it was expected that cations would permeate the GFETs. To determine whether cations accumulated around the graphene, an XPS analysis was performed on the graphene before and after immersion in the electrolyte solution. Two GFET substrates were prepared as samples for the XPS evaluation. After the GFETs were fabricated, one substrate was immersed in 0.1× D-PBS(-) for 25 h, while the other was not. Finally, both substrates were rinsed with deionized water, and the water was blown with N_2_ gas and removed on a hot plate at 120 °C.

**Figure 11:**
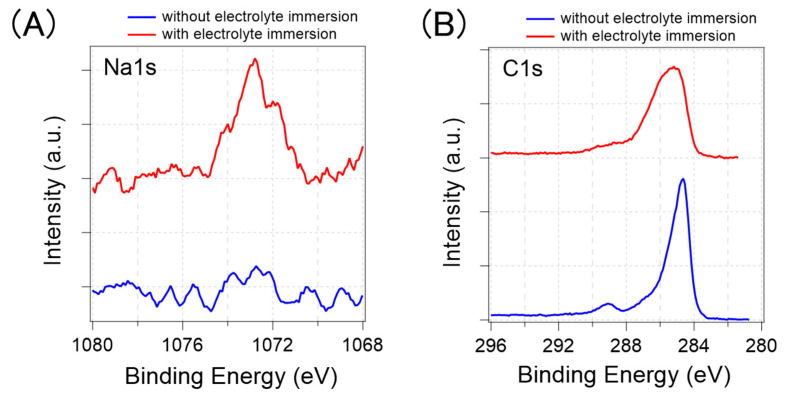
XPS analysis results of graphene with (red line) and without the electrolyte immersion (blue line). (**A**) Na1s peak (**B**) C1s peak.

Figure 11 shows the XPS analysis results for the Na1s and C1s. Figure 11A shows that the sodium-derived signals were detected only from the GFETs immersed in D-PBS(-), which indicates that the GFETs absorbed cations during the immersion. The relative atomic concentration of Na to Carbon was calculated at 3%, considering the peak area of the Na1s and C1s. However, from the XPS analysis, it was difficult to understand where sodium accumulated because of the low spatial resolution and low intensity of the Na1s. Some reports have shown that water molecules penetrate between graphene and substrates [31,32] and into PMMA resin [35]. Therefore, we expected that sodium would be located in these places, together with water molecules, as positively charged sodium ions may be adsorbed on the negatively charged substrate surface and PMMA. In addition, cation–π interactions should also facilitate the adsorption of Na^+^ onto the graphene’s surface [33,34]. Furthermore, based on the results, Na^+^ as well as other cations, such as K^+^, should be adsorbed.

Figure 11B shows the peak of the C1s. The sp^2^ C–C bonding in graphene is assigned at approximately 285 eV, and some other tails appearing on the high-energy side correspond to the binding energy of species in PMMA or functional groups on graphene [36]. In the case of the GFET without electrolyte immersion, the graphene-derived 285 eV peak was more prominent than the PMMA-derived 289 eV peak; thus, the surface of the GFET was less contaminated with PMMA. The GFET with electrolyte immersion has a wider C1s peak, especially on the high-energy side, compared to those without electrolyte immersion. This result implies that C–C bonds of graphene partly become C–O bonds during electrolyte immersion. In the GFETs, the graphene and gold electrodes were electrically connected with the electrolyte solution. Under such circumstances, a galvanic current may flow through the graphene, gold electrodes, and electrolytes, which occurs when two different metals are present in an electrolyte solution. The current possibly caused a redox reaction on the graphene surface [37]. Consequently, the graphene may have been partially oxidized. This oxidation of the graphene could also have contributed to the change in the electrical properties, although the transfer curves were not significantly changed by the pre-treatments, aside from the CNP shift.

## 4. Conclusions

We developed GFETs and evaluated the changes in their electrical characteristics. The GFETs using CVD graphene sheets and fabricated on SiO_2_ were highly p-doped, which was confirmed by the positive values of CNP. There are two main origins of p-doping: (i) PMMA residue and (ii) SiO_2_ substrates. The p-doping seemed to be gradually countered; that is, the CNP became close to zero during the electrical measurements over time. We proposed a possible mechanism for the drift, in which the two origins of p-doping were gradually countered by cations, such as the K^+^ and Na^+^ included in the D-PBS(-). This mechanism was supported by the experimental results: (i) when the GFETs were sufficiently countered with cations, which are known as n-dopants to graphene, the transfer curves were stable during long-term electrical measurements, and (ii) cations were detected in the GFETs after immersion in the electrolyte solution. Finally, by countering the GFETs with cations prior to evaluation, the CNP drift was suppressed by 96%. Our method should produce a stable GFET sensor platform.

Further experiments are needed to comprehensively understand the mechanism of cation accumulation in graphene. It might be possible to discover a more effective pre-treatment method by studying the mechanism in more detail. Furthermore, the functionalization of graphene sensors is possible by controlling the amount of accumulated cations.

## Data Availability

All data collected in this study are presented in this article.
